# A Chromosome-Scale Assembly of the Asian Honeybee *Apis cerana* Genome

**DOI:** 10.3389/fgene.2020.00279

**Published:** 2020-03-27

**Authors:** Zi-Long Wang, Yong-Qiang Zhu, Qing Yan, Wei-Yu Yan, Hua-Jun Zheng, Zhi-Jiang Zeng

**Affiliations:** ^1^Honeybee Research Institute, Jiangxi Agricultural University, Nanchang, China; ^2^Shanghai-MOST Key Laboratory of Health and Disease Genomics, Chinese National Human Genome Center at Shanghai, Shanghai, China

**Keywords:** *Apis cerana*, genome assembly, Hi-C, SMRT, chromosome-scale

## Abstract

*Apis cerana* is one of the main honeybee species in artificial farming, which is widely distributed in Asian countries. The genome of *A. cerana* has been sequenced by several different research groups using second generation sequencing technologies. However, it is still necessary to obtain more complete and accurate genome sequences. Here we present a chromosome-scale assembly of the *A. cerana* genome using single-molecule real-time (SMRT) Pacific Biosciences sequencing and high-throughput chromatin conformation capture (Hi-C) genome scaffolding. The updated assembly is 215.67 Mb in size with a contig N50 of 4.49 Mb, representing an 212-fold improvement over the previous Illumina-based version. Hi-C scaffolding resulted in 16 pseudochromosomes occupying 97.85% of the assembled genome sequences. A total of 10,741 protein-coding genes were predicted and 9,627 genes were annotated. Besides, 314 new genes were identified compared to the previous version. The improved high-quality *A. cerana* reference genome will provide precise sequence information for biological research of *A. cerana*.

## Introduction

*Apis cerana* is one of the main honeybee species that can be systematically raised by human beings and has been widely cultivated in the eastern countries such as China, Japan, and India for a long time, bringing considerable economic benefits to beekeepers. Compared with western honeybees, *A. cerana* is more sensitive to smell, better at using sporadic honey sources, and more suitable for collecting a variety of honey plants, while western honeybees usually prefer to collect large single honey source. Moreover, the *A. cerana* has stronger disease and stress resistance, stronger cold tolerance, lower feed consumption and longer collection period compared to western honeybees (Chen, 2001).

Genome sequences are of great significance to the basic biological research of a species. The first genome assembly of *A. cerana* of 228.32 Mb was reported by a Korean research group in 2015 (Park et al., 2015). Then, in 2017 our research group reported the genome assembly of the Chinese native species *A. cerana* cerana, with genome size of 228.79 Mb (Diao et al., 2018). Also, the genome assembly of another important eastern honeybee subspecies *A. cerana* japonica of 211.20 Mb is available in Genbank database (NCBI GCA_002217905.1).

The third generation sequencing (TGS) technologies do not need PCR amplification, which can effectively avoid systematic errors caused by bias of PCR amplification. At the same time, the lengths of DNA sequence fragments sequenced from a single run are so long that the average length of the reads can reach 10,000 bp, which is very helpful for assembling repetitive sequences. Compared with the second generation sequencing technologies, the TGS technologies also maintain the advantages of high throughput and low cost. Typical TGS technologies are single-molecule real-time (SMRT) sequencing technology developed by Pacific Biosciences company and Nanopore sequencing technology launched by Oxford Nanopore company, which are now widely used in genome sequencing of animals and plants (Jiang et al., 2014; Jiao et al., 2017; Gong et al., 2018; Kronenberg et al., 2018; Ghurye et al., 2019; Low et al., 2019; Wallberg et al., 2019; Zhang et al., 2019).

Here we used PacBio and Hi-C technologies to generate a highly contiguous *de novo* assembly of the eastern honeybee, *A. cerana*. The PacBio long reads from haploid drones were assembled into contigs, then, they were further assembled into scaffolds using Hi-C proximity ligation data. The completeness and contiguity of this new assembly was greatly improved compared to previous genome assemblies.

## Materials and Methods

### Library Construction and Sequencing

The experimental bees used in this study were 6-day old drone pupae sampled from a single colony in a *A. cerana* apiary raised by a local bee keeper in Fulong Township, Baisha Li Nationality Autonomous County, Hainan province (19°22′26″N, 109°28′20″E), China, in October 2018. The intestines of the pupae were removed to avoid contamination of gut microbes before construction of SMRTbell and Hi-C libraries. Two SMRTbell libraries were constructed and sequenced. For each library, DNA was extracted from a single intestine removed drone pupa by AxyPrep^TM^ Multisource Genomic DNA Miniprep Kit (Axygen, United States). Genomic DNA concentration was measured using the Qubitfluorimetry system with the High Sensitivity kit for detection of double-stranded DNA (Thermo Fisher Scientific, United States). Fragment size distribution of the genomic DNA was assessed using the Agilent 2100 Bioanalyzer with the 12000 DNA kit (Agilent, United States). Then, 5 μg of high molecular weight genomic DNA was sheared using g-Tube (Covaris, United States) to 10 kb, and the sheared DNA was used as input into the SMRTbell library preparation. SMRTbell library was prepared using PacBio 10 kb library preparation protocol. Once library was completed, it was size selected from 10 kb using the Blue Pippin instrument (Sage Science, United States) to enrich for the longest insert size. The library was sequenced on a PacBio Sequel system using Sequencing Kit 3.0, 1200 min movies with 120 min pre-extension and software v6.0 (PacBio).

One Hi-C library was created from a single intestine removed drone pupa of *A. cerana*. Briefly, the drone pupa was fixed with formaldehyde for 10 mins and then was lysed in lysing solution (500 μL 10 mM Tris–HCl pH 8.0, 10 mM NaCl, 0.2% Igepal CA-630 and 50 μL protease inhibitors). Then, the cross-linked genomic DNA was digested with *Hin*dIII overnight. Sticky ends of the genomic DNA were biotinylated and proximity-ligated to form chimeric junctions that were enriched for and then physically sheared to a size of 300–700 bp. Chimeric fragments representing the original cross-linked long-distance physical interactions were then processed into paired-end sequencing library, and sequenced on the Illumina HiSeq X Ten platform.

### Genome Assembly

After PacBio sequencing and removal of low quality reads, the remaining PacBio clean reads were assembled into contigs using HGAP4 of SMRT Link (version 6.0) (Chin et al., 2013). To assemble contigs into scaffolds, long-range contact reads were generated by Hi-C sequencing and adapter sequences of raw reads for Hi-C sequencing were trimmed and low quality PE reads were removed. Then the raw contigs were split into segments of 50 kb on average. The Hi-C clean data were mapped to these segments using BWA (version 0.7.10-r789) (Li and Durbin, 2009). The uniquely mapped pair-end reads were retained to perform assembly by using LACHESIS software (Burton et al., 2013). Any two segments which showed inconsistent connection with information from the raw contigs were checked manually. These corrected contigs were then assembled with LACHESIS.

### Repetitive Sequences and Genome Annotation

The RepeatModeler (version 1.73; RepeatModeler, RRID: SCR 015027) (Smit and Hubley, 2008) and RepeatMasker (version 3.3.0; RepeatMasker, RRID: SCR 012954) (Chen, 2004) were used to detect repeat sequences in the assembled genome. Gene prediction was performed using Augustus (version 3.3.2) (Stanke et al., 2006) with an *A. mellifera* model and known *A. cerana* genes as input. First, known *A. cerana* genes were mapped to the new genome assembly by BLAT v35 (Kent, 2002), then the Augustus scripts were used to create a hints file. *De novo* gene structure identification was performed using Augustus. The functional annotation of predicted genes was performed through BlastP against the NCBI non-redundant peptide database (NR), with parameters setting at E-value 1e-5. Gene ontology analysis was performed using Blast2GO (Conesa et al., 2005) through BlastP against the Swiss-Prot database (Bairoch and Apweiler, 2000) with a parameter of E-value 1e-5. Protein motif and KOG assignment was predicted through RPS-BLAST with the Conserved Domain Database (CDD) (Marchler-Bauer et al., 2011) with E-value 1e-5. The metabolic pathway was constructed based on the KEGG database (Kanehisa and Goto, 2000) by BBH method.

### Evaluation of the Genome Quality

The quality of the assembled genome and annotated gene sets were assessed first using the Bench marking Universal Single-Copy Orthologs (version 3.1.0; BUSCO, RRID: SCR_015008) (Simão et al., 2015) with the hymenoptera_odb9 dataset. To further assess the completeness of the predicted genes, we used three Illumina RNA-seq data from our previous study of the genome v2.0 (Diao et al., 2018) to map the predicted gene sets of genome assembly v2.0 and v3.0, respectively, using bowtie2 software (Langmead and Salzberg, 2012) with default parameters. In this study, we named the previous genome assembly of *A. cerana* reported by us as genome version 2.0, and this new assembly as version 3.0.

### Genome Comparison

Comparison of the predicted genes between genome v2.0 and v3.0 was performed using BlastP program with default parameters. The transcripts of the identified new genes were analyzed by mapping RNA-seq data from *A. cerana* in our previous research (Diao et al., 2018) to the predicted genes of genome v3.0 using bowtie2 software. Gene fusion and splitting between genome v2.0 and v3.0 were detected by sequence alignment of all the predicted CDSs of these two genomes using BlastN program with E-value 1e-100.

Collinearity analysis between *A. cerana* genome v3.0 and the latest genome sequences of *A. mellifera* (version Amel_HAv3.1)^1^ was performed using Mummer (Delcher et al., 2003) software with default parameters. Comparison of the domains of all the predicted genes between *A. cerana* genome v3.0 and the *A. mellifera* genome Amel_HAv3.1 was conducted in the Pfam database (El-Gebali et al., 2019) by RPS-BLAST with E-value 1e-3. Unique genes in *A. cerana* and *A. mellifera* were analyzed using BlastP program with E-value 1e-3.

## Results and Discussion

### Genome Sequencing and Assembly

After PacBio sequencing, 1,379,634 clean reads with total size of 28,919,033,693 bp, N50 of 38,250 bp and mean length of 20,961 bp were obtained (Table 1). The 28.92 Gb PacBio clean reads were assembled into 200 contigs with size ranging from 3,587 bp to 11,106,448 bp using HGAP4 of SMRT Link (version 6.0) (Chin et al., 2013; Table 2). The total size of the assembled contigs is 215,661,233 bp with average length of 20,961 bp and N50 of 4,485,954 bp, which represents an about 212-fold improvement in completeness contiguity compared to genome v2.0 of *A. cerana*.

**TABLE 1:** Summary of sequencing data for the new assembly of *A. cerana* genome.

	**PacBio**	**Hi-C**
Number of bases	28,919,033,693	42,964,780,942
Number of reads	1,379,634	143,436,579
N50 read length (bp)	38,250	150
Mean reads length (bp)	20,961	150

**TABLE 2:** Statistics of the Pacbio and Hi-C assembly.

	**Contig**	**Scaffold**
Assembly length (bp)	215,661,233	215,670,033
Number	200	126
N50 (bp)	4,485,954	13,422,783
Largest (bp)	11106448	26,804,424

**FIGURE 1:**
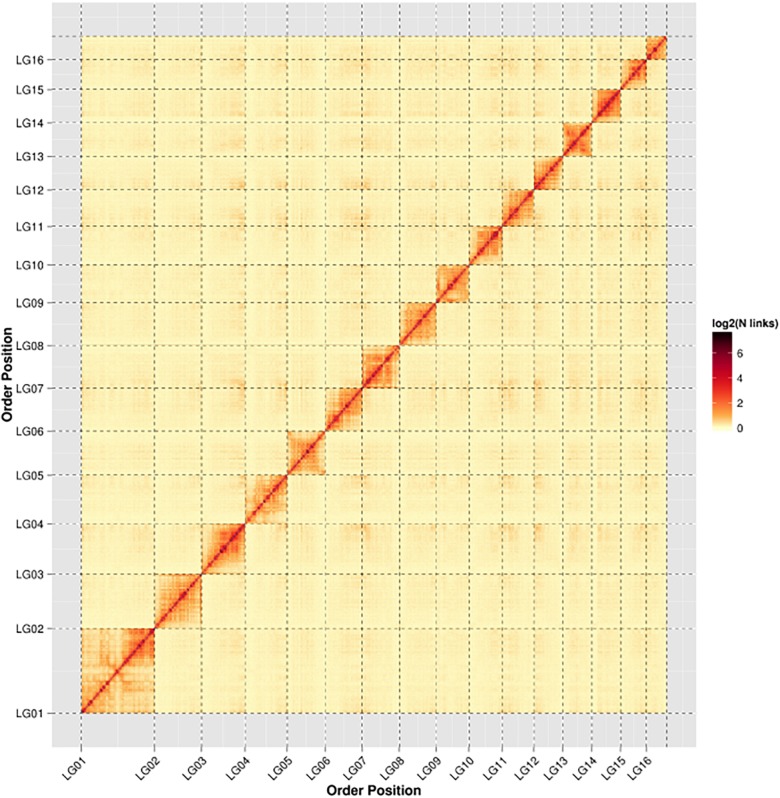
Heat map of Hi-C contact information of the 16 chromosomes. Pixel colors represent different normalized counts of Hi-C links between 50-kb non-overlapping windows for all 16 chromosomes (chr) on a logarithmic scale.

In order to assemble contigs into scaffolds further, 143,436,579 Hi-C reads with total size of 42.96 Gb were generated through Illumina HiSeq X Ten platform (Table 1), and they were used to link the 200 contigs into 126 scaffolds with an N50 length of 13.42 Mb (Table 2 and Supplementary Table S1). The final scaffolds contain 16 pseudomolecules representing the 16 chromosomes of *A. cerana* (Figure 1) and 110 unplaced scaffolds. The total length of the 16 pseudochromosomes with length range from 7.10 Mb to 26.80 Mb is 211.03 Mb, consisting of 97.85% of the whole genome sequences (Supplementary Table S1). The mean length of the remaining 110 unplaced scaffolds is 0.04 Mb which is far below 13.19 Mb of mean length of the 16 pseudochromosomes, suggesting these unplaced scaffolds may consist mainly of repetitive sequences.

### Genome Annotation and Evaluation

Repeat sequences are widely distributed in higher organisms and play an important role in regulation of gene transcription (Fotsing et al., 2019; Wu et al., 2019), DNA replication (Madireddy and Gerhardt, 2017), and so on. We analyzed categories and proportions of repeat sequences in this new assembly. Finally, 19,731,355 bp of repeat sequences were detected accounting for 9.15% of the genome, which is significant higher than the 14.79 Mb (6.48%) in the assembly reported by Korean scientists (Park et al., 2015) and the 9.61 Mb (4.2%) in the assembly v2.0 reported by us (Diao et al., 2018), indicating that third generation sequencing technologies are beneficial to identification of repeat sequences. These repeat sequences include long interspersed nuclear elements (LINEs) (0.04%), long terminal repeats (LTRs) (0.20%), DNA elements (0.65%), small RNAs (0.01%), simple repeats (4.03%), low complexity sequences (1.06%), and unclassified repeat sequences (3.16%). Of them, simple repeats with total length of 8,693,708 bp are the richest type (Table 3), which is consistent with the previous reports (Park et al., 2015; Diao et al., 2018) and is the same as in *A. mellifera* (Wallberg et al., 2019), suggesting that simple repeats are the major type of repeat sequences in honeybees.

**TABLE 3:** Transposable elements and repeat sequence statistics.

**Classification**	**Number of elements**	**Length (bp)**	**Percentage (%)**
LINEs	786	84,104	0.04
LTR elements	983	441,743	0.20
DNA elements	5,809	1,393,124	0.65
Unclassified	24,299	6,823,093	3.16
Small RNA	45	31,168	0.01
Simple repeats	191,534	8,693,708	4.03
Low complexity	43,237	2,285,374	1.06

A total of 10,741 protein-encoding genes were predicted, with an average gene size of 5,069 bp and an average coding DNA sequence (CDS) size of 1,519 bp. The average exon and intron sizes were 247 bp and 688 bp, respectively. 9,627 genes occupying 89.63% of the whole set of predicted genes were annotated through KEGG, KOG, Pfam and uniprot databases (Table 4).

**TABLE 4:** General statistics of the functional annotation.

	**Database**	**Number**	**Percentage (%)**
Total		10,741	100
	KEGG	9,303	86.61
	KOG	7,629	71.03
	Pfam	7,776	72.40
	Uniprot	4,884	45.47
Unannotated		1,114	10.37

The quality of the assembled genome and annotated gene sets were assessed using BUSCO. Among the 4,415 BUSCO groups searched, 4,116 BUSCO groups (including complete and fragmented BUSCOs) were identified, occupying 93.23% of the total BUSCO groups. Among them, 3,801 groups were complete BUSCO groups, occupying 86.09% of the total BUSCO groups supporting the high quality of the genome assembly (Table 5).

**TABLE 5:** Statistics of the BUSCO assessment.

	**BUSCO groups**	**Percentage (%)**
Complete BUSCOs	3,801	86.09
Complete and single-copy BUSCOs	3,792	85.89
Complete and duplicated BUSCOs	9	0.20
Fragmented BUSCOs	315	7.13
Missing BUSCOs	299	6.77
Total BUSCO groups searched	4,415	100

The completeness of the predicted genes was further assessed by mapping Illumina RNA-seq data from our previous study (Diao et al., 2018) to the predicted gene sets of genome v2.0 and v3.0, respectively, which results in 31.27–41.59% of the reads mapping to gene sets of genome v3.0, while the ratio is just 20.66–24.83% when gene sets of genome v2.0 was used as mapping reference (Supplementary Table S2). It suggests that the completeness of the predicted genes is significantly improved in the novel assembly of *A. cerana* genome.

### Comparison Between Genome v2.0 and v3.0

We compared this new genome assembly with the previous version of *A. cerana* genome reported by us (Diao et al., 2018). Compared to genome v2.0, the size of this improved genome was significantly reduced, which mainly due to the elimination of a large number of “N” representing sequencing gaps that exist in genome v2.0 (there are a total of 19.55 Mb “N” in the genome v2.0) (Supplementary Table S3).

We compared the predicted genes between genome v2.0 and v3.0, a total of 314 new genes were identified in genome v3.0 (Supplementary Table S4). Of them, 154 genes are newly assembled in genome v3.0 that their nucleotide sequences do not exist in genome v2.0, and 160 genes are newly predicted in genome v3.0 while not predicted in v2.0 because of sequencing gaps, assembling errors and so on. The mean length of the CDS region of all the newly predicted genes is just 321 bp which is significantly shorter than that of all the predicted genes, suggesting that most of the identified new genes encode proteins with small molecular weight, but the mean length of the introns of the 314 new genes is significantly longer than that of all the predicted genes (Supplementary Figure S1). 268 of the 314 new genes (85.35%) are annotated as “hypothetical protein” or “uncharacterized conserved protein,” suggesting that most of the new genes are functionally unknown. Of the genes with definite functions, genes annotated as “Predicted ubiquitin-protein ligase/hyperplastic discs protein, HECT superfamily,” “HIV-1 Vpr-binding protein” and “Dosage compensation complex, subunit MLE” have more than 10 copies in the genome, suggesting that third generation sequencing technologies have greatly improved success rate of identifying multi copy genes during whole genome sequencing which is very difficult using second generation sequencing technologies. Besides, RNA polymerase I large subunit (g2476.t1), ATP-dependent RNA helicase (g4187.t1), nuclear protein Ataxin-7 (g7042.t1), and so on, are important functional genes in the new gene list. We found that 164 new genes have transcription evidence when mapping RNA-seq data from *A. cerana* to the predicted genes of genome v3.0, implying that most of the new genes identified in genome v3.0 are really existed and have transcriptional activity in *A. cerana*.

We detected gene fusion and splitting between genome v2.0 and v3.0. The results showed that 78 genes in genome v2.0 were fused into 38 genes in genome v3.0; on the other hand, 135 genes in genome v2.0 each was spitted into more than two genes in genome v3.0 (Supplementary Table S5).

### Genome Comparison Between *A. cerana* and *A. mellifera*

**FIGURE 2:**
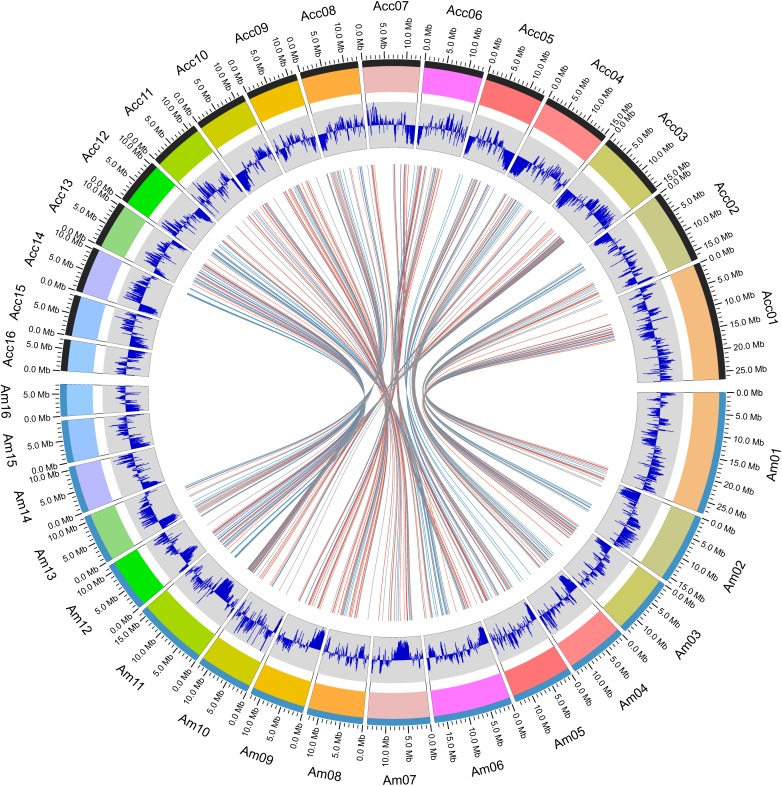
The atlas of the *A. cerana* chromosomes.

Within the genus *Apis* species, *A. cerana* and *A. mellifera* have the closest relationship. Their niche overlaps and competition between these two species is fierce, especially the drones of *A. mellifera* can interfere with the mating behavior of *A. cerana* queens and drones resulting in decline of the *A. cerana* colonies year by year. We compared the collinearity between *A. cerana* genome v3.0 and the *A. mellifera* genome Amel_HAv3.1 (Figure 2 and Supplementary Figure S2). The 16 pseudochromosomes we identified in the new assembly of *A. cerana* genome aligned exactly against the 16 chromosomes of the *A. mellifera* genome Amel_HAv3.1 with high similarity. For the 16 chromosomes, chromosomes 1, 2, 5, 6, 9, 15 show almost completely collinear between the *A. cerana* genome v3.0 and *A. mellifera* genome Amel_HAv3.1; and chromosome 3, 4, 10, 13, 14, 16 just have small stretches of non-collinear region; for chromosome 7, 8, 11, 12, the collinear lines are split into several fragments because of large chromosome inversion, but in each fragment it is highly collinear between *A. cerana* and *A. mellifera*. These results suggest that our assembly is of high continuity compared with the *A. mellifera* genome.

We compared the domains of all the predicted genes between *A. cerana* genome v3.0 and the *A. mellifera* genome Amel_HAv3.1. A total of 4,026 Pfam family domains existed in both *A. cerana* and *A. mellifera* (Supplementary Table S6). The total number of genes containing Pfam family domains showed no obvious difference between *A. cerana* and *A. mellifera*. Among the identified Pfam family domain containing genes, the number of “7 tm Odorant receptor” (PF02949) domain containing genes showed great difference between *A. cerana* and *A. mellifera*, 63 genes vs. 137 genes, which is consistent with the results reported in the previous two genome assemblies of *A. cerana* (Park et al., 2015; Diao et al., 2018), suggesting that there are significant differences in olfactory ability between these two species. Besides, 43 Pfam family domains are unique to *A. cerana*, and 314 Pfam family domains are unique to *A. mellifera*.

In addition, we analyzed unique genes in *A. cerana* and *A. mellifera*. Finally, 111 genes unique to *A. mellifera* and 71 genes unique to *A. cerana* were identified (Supplementary Table S7). Of the *A. mellifera* unique genes, 55 genes have KOG annotation, and “Signal transduction mechanisms” “General function Translation” are the major KOG categories. For *A. cerana* unique genes, most of them are annotated as “hypothetical protein,” just 30 genes have KOG annotation and half of them belong to “transcription” category, suggesting that these genes might promote the formation of unique characters of *A. cerana* by regulating gene transcription.

## Conclusion

We reported the newly assembled genome of *A. cerana* using an integrated strategy of PacBio and Hi-C technologies. This new genome assembly has an about 212-fold improvement in contiguity over the previous version based on second generation sequencing technologies, and many new genes were identified. This genome assembly will be an important genome reference for studies of functional genes in *A. cerana*. Also, it will provide important data resource for evolutionary and comparative genomic studies.

## Data Availability Statement

The genome assembly 3.0 of *A. cerana* data has been submitted to the NCBI under Bio-Project number PRJNA579740. The Hi-C and PacBio sequencing data are available from NCBI via accession numbers SRR10377217, SRR10377218, and SRR10377219.

## Author Contributions

Z-JZ and H-JZ conceived and designed the research. Z-LW and W-YY prepared the experimental materials. Y-QZ conducted the experiments. Y-QZ, H-JZ, QY, and Z-LW analyzed the data. Z-LW and Y-QZ wrote the manuscript. All authors read and approved the publication of the manuscript.

## Conflict of Interest

The authors declare that the research was conducted in the absence of any commercial or financial relationships that could be construed as a potential conflict of interest.

## Footnotes

1 ftp://ftp.ncbi.nlm.nih.gov/genomes/Apis_mellifera/

## Supplementary Material

FIGURE S1 Length of the CDS, exons and introns of newly identified genes compared to those of all the predicted genes.

FIGURE S2 Colinearity of chromosomes between *A. cerana* and *A. mellifera*. The *x*-axis represents the chromosomes from *A. mellifera*, *x*-axis represents the corresponding chromosomes from *A. cerana*.

TABLE S1 The list of the 200 contigs and the final 126 scaffolds assembled from contigs.

TABLE S2 Mapping of RNA-seq reads to the predicted gene sets of genome v2.0 and v3.0.

TABLE S3 Comparison of the v2.0 and v3.0 assemblies of *A. cerana* genome.

TABLE S4 The list of all the newly identified genes in the genome v3.0 of *A. cerana*.

TABLE S5 The list of genes that undergo gene fusion and splitting between genome v2.0 and v3.0 of *A. cerana*. Based on the alignments of genome v2.0 and v3.0, 78 genes in genome v2.0 were fused into 38 genes in v3.0, 135 genes in genome v2.0 each was spitted into more than two genes in v3.0.

TABLE S6 The list of Pfam family domains identified in *A. cerana* and *A. mellifera*.

TABLE S7 The list of unique genes in *A. cerana* and *A. mellifera*.
