# Supplementary figures and images for: A Chromosome-Scale Assembly of the Asian Honeybee Apis cerana Genome
Source: Front Genet. 2020 Mar 27;11:279. doi: 10.3389/fgene.2020.00279 (PMC7119468; doi:10.3389/fgene.2020.00279)

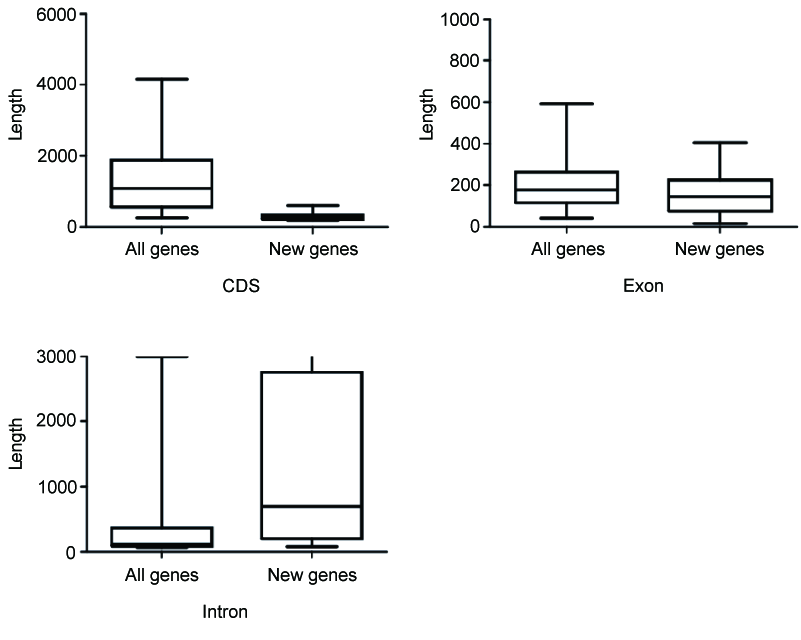

Supplement: FIGURE S1 — Length of the CDS, exons and introns of newly identified genes compared to those of all the predicted genes. [file Image_1.TIF]

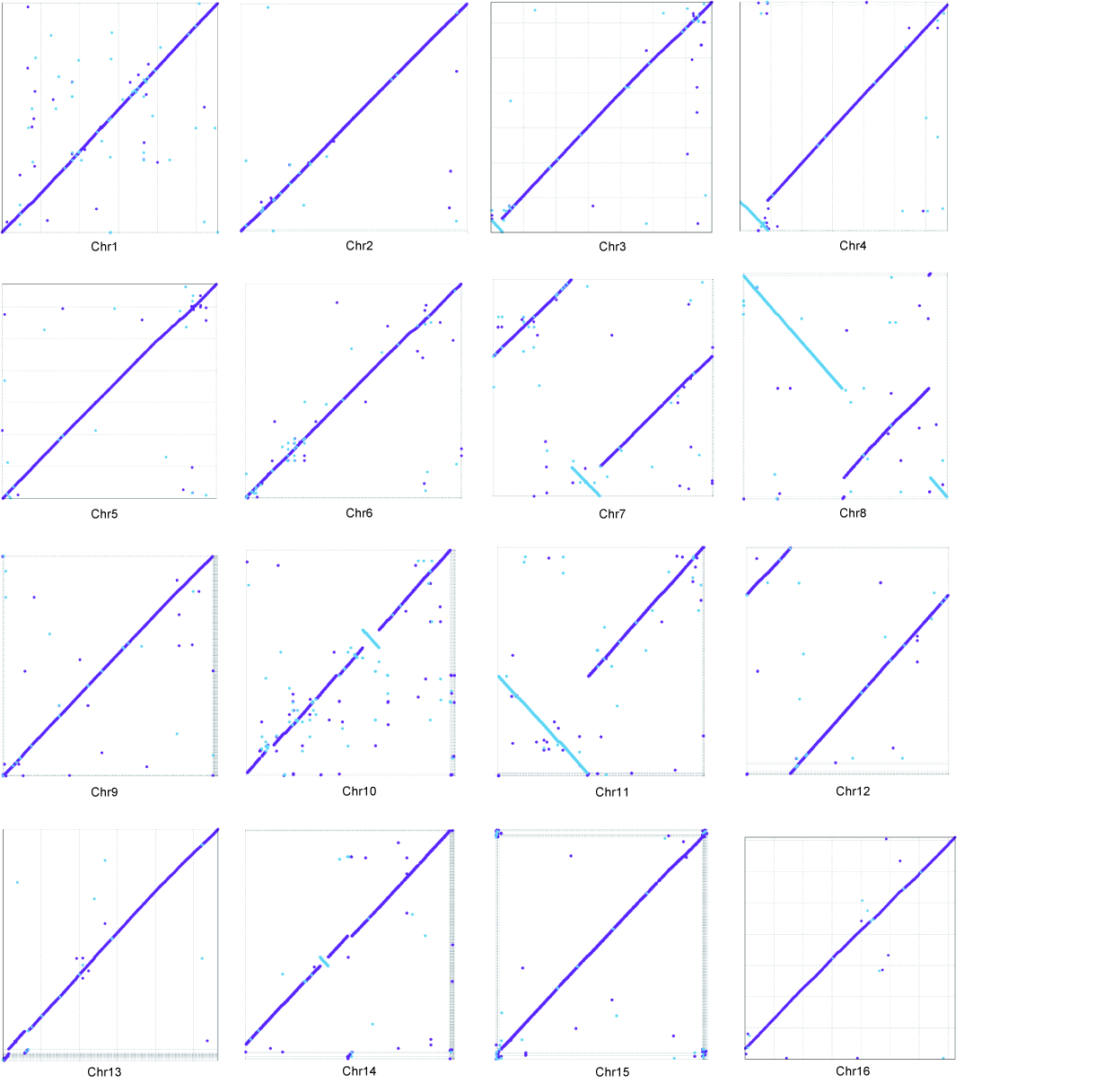

Supplement: FIGURE S2 — Colinearity of chromosomes between A. cerana and A. mellifera. The x-axis represents the chromosomes from A. mellifera, x-axis represents the corresponding chromosomes from A. cerana. [file Image_2.TIF]
