# Supplementary material for: A Chromosome-Scale Assembly of the Asian Honeybee Apis cerana Genome
Source: Front Genet. 2020 Mar 27;11:279. doi: 10.3389/fgene.2020.00279 (PMC7119468; doi:10.3389/fgene.2020.00279)
Supplement: TABLE S2 — Mapping of RNA-seq reads to the predicted gene sets of genome v2.0 and v3.0. [file Table_2.DOCX]

Table S2 Mapping of RNA-seq reads to the predicted gene sets of genome v2.0 and v3.0

|  | Gene sets of v2 genome | | |  | Gene sets of v3 genome | | |
| --- | --- | --- | --- | --- | --- | --- | --- |
|  | AC_CK | AC_clear | AC_shaking |  | AC_CK | AC_clear | AC_shaking |
| Total reads | 7416173 | 10890693 | 15690593 |  | 7416173 | 10890693 | 15690593 |
| Total mapped | 1841684 | 2249630 | 3576818 |  | 3084176 | 3405251 | 5631451 |
| Ratio of total mapped reads | 24.83% | 20.66% | 22.80% |  | 41.59% | 31.27% | 35.89% |
| Total unmapped reads | 5574489 | 8641063 | 12113775 |  | 4331997 | 7485442 | 10059142 |
| Ratio of total unmapped reads | 75.17% | 79.34% | 77.20% |  | 58.41% | 68.73% | 64.11% |
