# Supplementary material for: A Chromosome-Scale Assembly of the Asian Honeybee Apis cerana Genome
Source: Front Genet. 2020 Mar 27;11:279. doi: 10.3389/fgene.2020.00279 (PMC7119468; doi:10.3389/fgene.2020.00279)
Supplement: TABLE S3 — Comparison of the v2.0 and v3.0 assemblies of A. cerana genome. [file Table_3.DOCX]

Table S3 Comparison of the v2.0 and v3.0 assemblies of *A. cerana* genome.

|  | v2.0 | v3.0 |
| --- | --- | --- |
| Total assembly size (bp) | 228,791,026 | 215,670,033 |
| Number of contigs | 21,784 | 200 |
| Number of scaffolds | 879 | 126 |
| Contig N50 (bp) | 21,160 | 4,485,954 |
| Scaffold N50 (bp) | 1,393,515 | 13,422,783 |
| Number of predicted genes | 10,182 | 10,741 |
